# Respiratory Infant Mortality Rate by Month of Birth in Mexico

**DOI:** 10.3390/epidemiologia6040083

**Published:** 2025-12-02

**Authors:** Alessandro Milán, Juan C. Cuevas-Tello, Daniel E. Noyola

**Affiliations:** 1 Centro de Investigación en Ciencias de la Salud y Biomedicina, Universidad Autónoma de San Luis Potosí, Av. Sierra Leona 550, San Luis Potosí 78210, San Luis Potosí, Mexico; alessandro.milan@uaslp.mx (A.M.); cuevas@uaslp.mx (J.C.C.-T.); 2 Facultad de Ingeniería, Universidad Autónoma de San Luis Potosí, Av. Dr. Manuel Nava 8, San Luis Potosí 78210, San Luis Potosí, Mexico; 3 Centro de Investigación y Estudios de Posgrado, Facultad de Ingeniería, Universidad Autónoma de San Luis Potosí, Av. Dr. Manuel Nava 8, San Luis Potosí 78210, San Luis Potosí, Mexico; 4 Facultad de Medicina, Universidad Autónoma de San Luis Potosí, Av. Venustiano Carranza 2405, San Luis Potosí 78210, San Luis Potosí, Mexico

**Keywords:** infant mortality, Acute Respiratory Infections, respiratory syncytial virus, seasonality, regional variations

## Abstract

Background: Respiratory syncytial virus (RSV) is a major contributor to severe Acute Respiratory Infections (ARI) in infants worldwide, leading to significant morbidity and mortality. The seasonal nature of RSV and other respiratory infections presents unique risks, especially for infants in low- and middle-income countries, such as Mexico, where comprehensive RSV surveillance is limited. This study aims to analyze respiratory infant mortality rates by month of birth across Mexico, with a focus on identifying high-risk periods and regional differences. Methods: National birth and mortality data from the Instituto Nacional de Estadística y Geografía were analyzed for all infants born between April 2014 and March 2020. Respiratory mortality rates (based on ICD-10 J and U codes) were calculated by month of birth and examined across eight geographical regions in Mexico. Mortality trends were analyzed using descriptive statistics to assess seasonal and regional variations. A correlation analysis was conducted between respiratory mortality and confirmed RSV hospitalization data to assess the temporal relationship between increased mortality and epidemic activity of this virus. Results: A total of 12,604,902 live births were recorded in Mexico during the study period, with 8805 infant deaths attributed to respiratory causes, resulting in a respiratory infant mortality rate of 0.7 deaths per 1000 births. Mortality rates exhibited strong seasonal patterns, with infants born between September and November at higher risk of respiratory death, peaking in October. The highest mortality rates were observed in the South region, while the lowest rates were in the Northeast. Conclusions: These findings highlight the importance of implementing preventive strategies in Mexico that are aligned with regional RSV seasonality. Timing preventive interventions with regional and seasonal mortality trends should enhance the cost-effectiveness and impact of RSV immunization programs, ultimately reducing infant mortality nationwide.

## 1. Introduction

Respiratory syncytial virus (RSV) is the leading cause of severe Acute Respiratory Infections (ARI) in infants, and it is estimated to cause one in 50 deaths of children < 5 years of age worldwide [1]. Preterm infants, those with bronchopulmonary dysplasia, and children with congenital heart disease are among those at increased risk for severe infection and death [2,3]. Because of the high morbidity associated with this infection, specific preventive interventions have been developed including monoclonal antibodies and a vaccine currently available for maternal immunization [4,5,6]. However, a major limitation for implementation of preventive programs, particularly in low- and middle-income countries, is the high cost of some of these products [7]. Because of this, targeting high-risk populations has been a strategy established in many countries in order to enhance the cost-effectiveness of immunization [8].

Hospitalizations and mortality associated with respiratory diseases and ARI show a seasonal pattern in most countries. Because RSV presents in yearly epidemics, particular interest has been placed on defining the virus seasonality in each country. This information is important in order to define during which months of the year RSV preventive programs, such as administration of the monoclonal antibody palivizumab or maternal immunization with RSV vaccines, should be established [9]. As such, the seasonal distribution of RSV circulation, along with the age of hospitalized and deceased infants at the time of infection, has helped to identify infants at higher risk for poor outcomes in many high-income countries, based on their age at the start of the RSV season or their birth month [10]. In contrast, many low- and middle-income countries lack nationally representative RSV surveillance information, as well as systematic use of RSV detection assays in children hospitalized with ARI. However, a correlation between RSV circulation and increased ARI-associated hospitalizations in infants has been described, suggesting that severe ARI diagnoses could be used as an alternative for identifying seasonal patterns in the circulation of this virus [11]. Of note, in addition to RSV, other pathogens including influenza virus, human metapneumovirus, SARS-CoV-2, *Streptococcus pneumoniae*, and *Bordetella pertussis* have been identified as causes of fatal ARI in Mexico [3,12,13,14,15,16].

RSV infections show a seasonal pattern in the majority of the regions in Mexico, where yearly epidemics occur between fall and winter, and the majority of infections occur between October and March [3,17,18]. In the present study, we established ARI-associated infant mortality rates based on the month of birth in the Mexican population for all infants born between April 2014 and March 2020, based on national birth and mortality registries. These results should be helpful to guide the implementation of RSV-specific preventive programs.

## 2. Materials and Methods

### 2.1. Data

The data used for this analysis were obtained from the Subsystem of Demographic and Social Information provided by Instituto Nacional de Estadística y Geografía (INEGI) in Mexico [19,20]. The datasets include birth and mortality registries at a national level for all infants born between April 2014 and March 2020. Birth records were used to determine the total number of live births each month, while death records of infants under one year of age were used to identify respiratory-associated mortality. The dataset also included information on the geographical location of each event (birth and death), allowing a more detailed analysis of mortality patterns across different regions of the country.

### 2.2. Data Extraction

The data extraction process was conducted using Python 3.13.5, a computer programming language, to retrieve and prepare the information needed for analysis. Python scripts filtered relevant data, including date of death, cause of death, birth date, age, and socio-demographic characteristics of parents, compiling this information into structured tables. These tables contained data on monthly births, respiratory mortality diagnoses, infant mortality rates by month of birth and occurrence, regional variations, and socio-demographic characteristics of parents. This structured dataset provided a comprehensive foundation for analyzing respiratory infant mortality patterns across regions, time periods, and demographic groups, enabling a thorough assessment of seasonal and regional trends.

The first step in the data extraction process was to download the yearly mortality records from INEGI, along with the birth records. These records were aggregated to create a single dataset for deaths and another for births. Mortality records for infants older than one year and those with an unknown year or month of death were excluded. For birth registries, only records of children whose mothers resided in one of the 32 Mexican states were included, ensuring that the data analyzed comes only from infants with permanent residency in Mexico.

Next, the year and month of both death and birth were extracted from the datasets, and the age of infants was determined accordingly. Socio-demographic characteristics of the parents were included to assess key factors that might influence mortality rates. The data were then aggregated monthly and at different geographic levels, including national, state, and regional, to provide a comprehensive view of trends. Finally, mortality rates were calculated for each aggregation level, and the resulting information was used to generate the plots and tables presented in the paper.

### 2.3. Data Analysis

Respiratory deaths were identified based on ICD-10 codes beginning with the letters J (respiratory system diseases) and U (COVID-19). For simplicity, figures referring to “(J + U)” represent the combined set of these respiratory causes. Respiratory infant mortality rate was calculated for infants born between April 2014 and March 2020 by month of birth in Mexico. Mortality rates were determined at the national level and further divided into eight geographical regions. The analysis included: (1) all births registered between April 2014 and March 2020, and (2) death records of children under one year of age who died between April 2014 and March 2021. Deaths of children born after 30 March 2020 were excluded from the analysis. The month of birth for each infant who died was obtained from mortality records, and the total number of deaths was divided by the total number of births in the corresponding month, multiplied by 1000.

Regional analyses were carried out dividing the country into eight regions, considering the wide geographical, climatological, and economical variations in Mexico [21]. This regional classification is based mostly on closeness between states, as well as geopolitical and economic factors. Use of a classification scheme based on biogeographic regions was not possible, since regions based on climate and vegetation do not follow state boundaries.

### 2.4. RSV Circulation and Respiratory Mortality

In order to assess the use of respiratory deaths as a proxy of RSV circulation pattern, we analyzed data from epidemiological studies carried out between 2002 and 2015 in San Luis Potosí (Mexico), since no systematic, year-round, nationally representative RSV surveillance data is currently available in Mexico. Data were obtained from several studies in which systematic, year-round testing for RSV in infants admitted to Hospital Central “Dr. Ignacio Morones Prieto”, Hospital del Niño y la Mujer “Dr. Alberto López Hermosa”, and Hospital General de Soledad de Graciano Sánchez was carried out [3,22,23]. These studies comprise the most comprehensive RSV epidemiological data obtained systematically on a specified population and during a prolonged period of time available in Mexico. We determined the monthly number of infants under one year of age hospitalized due to RSV infection identified in these studies and compared the seasonal distribution with that of infant mortality based on the month of death.

### 2.5. Statistical Analysis

A chi-square test of homogeneity was used to evaluate whether the distribution of respiratory infant mortality differed across months of birth, accounting for the number of live births in each month.

## 3. Results

### 3.1. Respiratory Infant Mortality Rate

Overall, there were 12,604,902 births between April 2014 and March 2020 in Mexico. Of these infants, 144,243 died before their first birthday, corresponding to an infant mortality rate of 11.44 deaths/1000 births; 8805 deaths were caused by respiratory illness, corresponding to a respiratory-infant mortality rate of 0.7 deaths per 1000 births. As such, respiratory deaths accounted for 6.1% of all deaths in infants during the first year of life. Most respiratory deaths (6531/8805; 74.1%) corresponded to Acute Respiratory Infections, including COVID-19; see Table 1.

**Figure 1:**
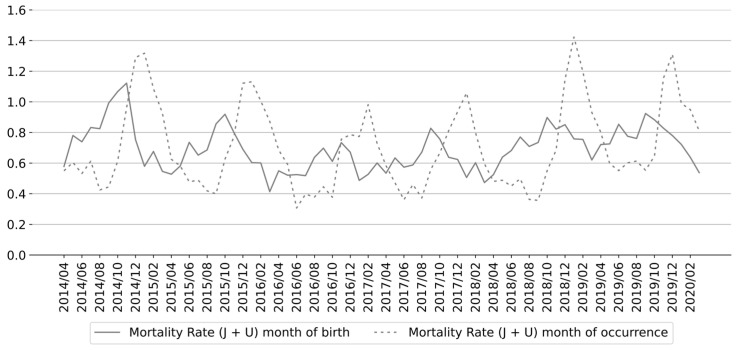
Monthly respiratory infant mortality rate in Mexico according to date of death and birth date between April 2014 and March 2020. (J + U) denotes respiratory deaths classified under ICD-10 codes beginning with the letters J (respiratory system diseases) and U (COVID-19).

**Figure 2:**
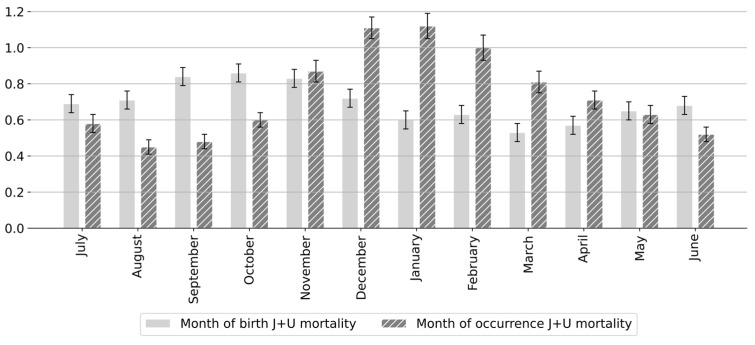
Average monthly respiratory (J and U ICD-10 codes) infant mortality rates based on month of occurrence and month of birth in Mexico (April 2014–March 2020). Error bars represent 95% confidence intervals for mortality rates based on observed counts. (J + U) denotes respiratory deaths classified under ICD-10 codes beginning with the letters J (respiratory system diseases) and U (COVID-19).

A chi-square test of homogeneity revealed that the distribution of respiratory infant deaths by month of occurrence was non-uniform (*χ*^2^(11) = 907.09; *p* < 0.001). The number of respiratory infant deaths showed a pronounced seasonal pattern, with peaks typically occurring in December or January of each winter season (Figure 1). Similarly, the distribution of deaths by month of birth differed significantly across the year (*χ*^2^(11) = 188.85; *p* < 0.001), with more deaths observed among infants born between August and December. The highest counts were recorded for those born in September, October, or November (Figure 1 and Figure 2). These results confirm a non-uniform seasonal pattern in respiratory infant mortality, consistent with increased respiratory infection activity during the colder months.

Respiratory mortality rates varied between states; see Table A1. The highest mortality rates occurred in Chiapas (1.40 deaths/1000 births), Puebla (1.03 deaths/1000 births), and Yucatan (1.01 deaths/1000 births), while the lowest mortality rates were registered in Morelos (0.32 deaths/1000 births), Colima (0.41 deaths/1000 births), and Queretaro (0.41 deaths/1000 births).

Regional analyses were carried out dividing the country into eight regions; see Table A2. The monthly mortality rates for each of the eight regions and countrywide are shown in Table 2. Respiratory mortality rates varied by region: the lowest mortality rate was recorded in the Northeast region (0.5 deaths/1000 births) and the highest in the South region (1.02 deaths/1000 births).

**Figure A1:**
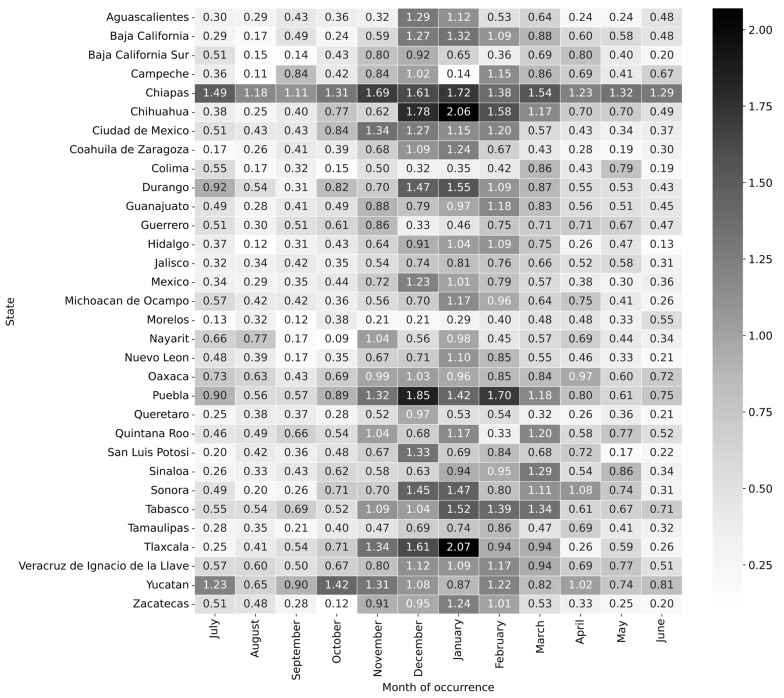
Monthly respiratory infant mortality rates (per 1000 live births) by state in Mexico, 2014–2020. The heatmap shows distinct seasonal patterns, with mortality rates increasing during winter months (December–January) and decreasing during spring and summer.

**Figure A2:**
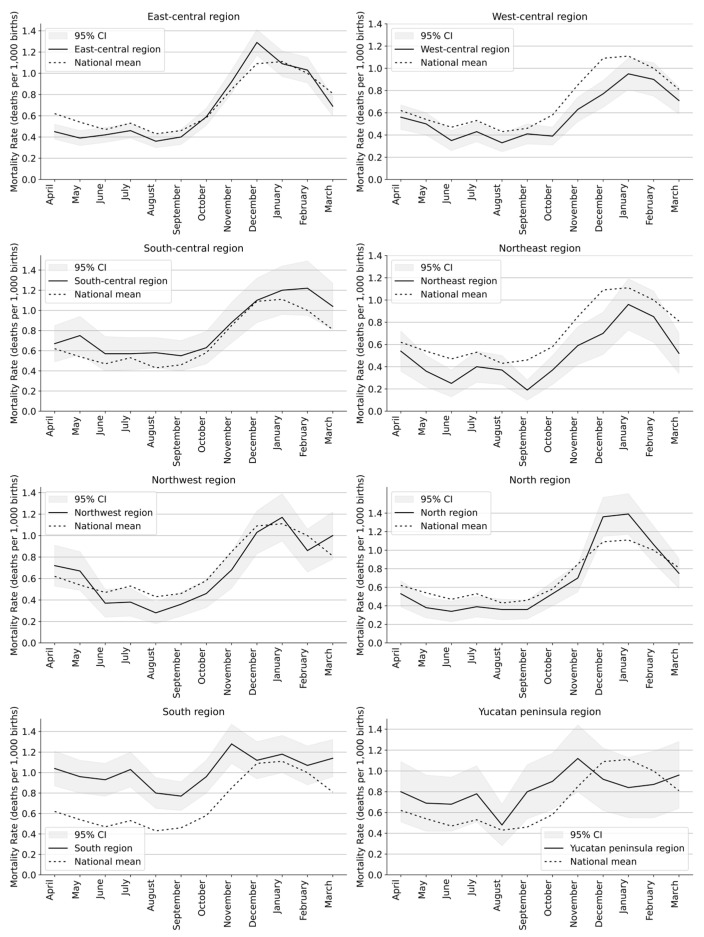
Respiratory mortality rates according to month of death for each region and nation-wide in Mexico for infants born between April 2014 and March 2020. Shaded bands represent 95% confidence intervals for mortality rates based on observed counts.

Figure A1 displays the monthly distribution of respiratory infant mortality rates across Mexican states. The results are consistent with the regional and state-level variations described above. Mortality rates increased during the winter months, typically peaking between December and January, and declined through the spring and summer. This seasonal pattern was observed nationwide but showed varying intensity across regions (Figure A2).

Overall, infants born in March had the lowest respiratory mortality rate (0.53 deaths per 1000 births), while those born in October had the highest (0.86 deaths per 1000 births). However, in the North region and the Yucatan Peninsula, infants born in April had the lowest mortality rates (0.41 and 0.45 deaths per 1000 births, respectively). Notably, the South region exhibited the least seasonal variation in mortality, with the lowest rates recorded for infants born in November (0.93 deaths per 1000 births), July (0.95 deaths per 1000 births), and January (0.95 deaths per 1000 births); nevertheless, even during those months, mortality rates were the highest in the country, well above the national average.

Infants born in October had the highest overall mortality rate (0.86 deaths per 1000 births). However, the month with the highest mortality varied by region. The highest mortality was recorded for infants born in September in three regions (Northwest, West-central, and South-central), for those born in October in two regions (East-central and Yucatan Peninsula), and for those born in November in two regions (North and Northeast). In the South region, the mortality rate was highest for infants born in May (1.21 deaths per 1000 births).

**Figure 3:**
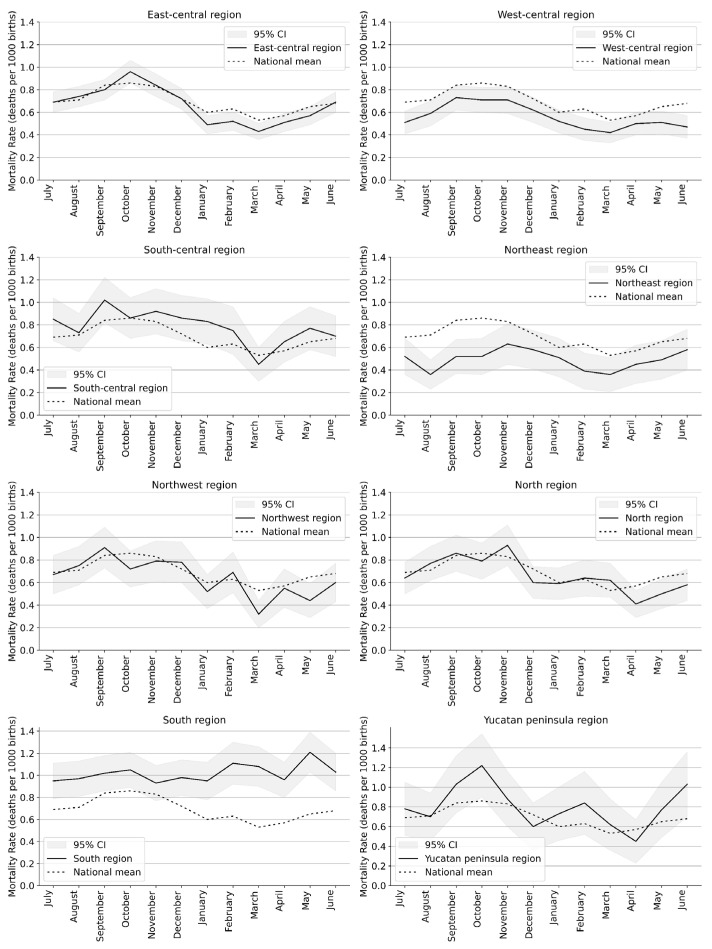
Respiratory mortality rates according to month of birth for each region and nationwide in Mexico for infants born between April 2014 and March 2020. Shaded bands represent 95% confidence intervals for mortality rates based on observed counts.

Figure 3 shows mortality rates by month of birth for each region compared to the national average. Mortality rates in the West-central and Northeast regions were lower than the national average, while rates in the North, Northwest, and East-Central regions were close to the average. In contrast, the South-Central, South, and Yucatan Peninsula regions had higher than average mortality rates.

To better understand factors influencing seasonality of infant mortality, we analyzed maternal and paternal characteristics of infants born during each month of the study period. No major differences were observed in maternal or paternal age, marital status, infants’ birth order, type of pregnancy (singleton or multiple birth), hospital birth, or maternal and paternal education across different months of birth; see Table A3 and Table A4.

### 3.2. Correlation Between Seasonal Respiratory-Associated Infant Mortality Rates and RSV Circulation Patterns

**Figure 4:**
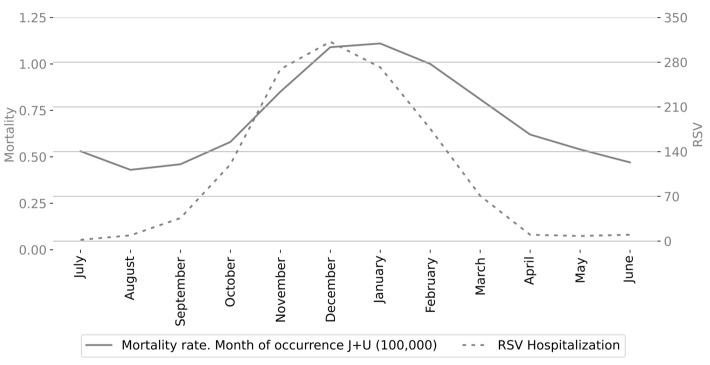
Monthly respiratory infant mortality rate in Mexico and total number of RSV-confirmed admissions of infants under one year of age in San Luis Potosí, Mexico.

**Figure 5:**
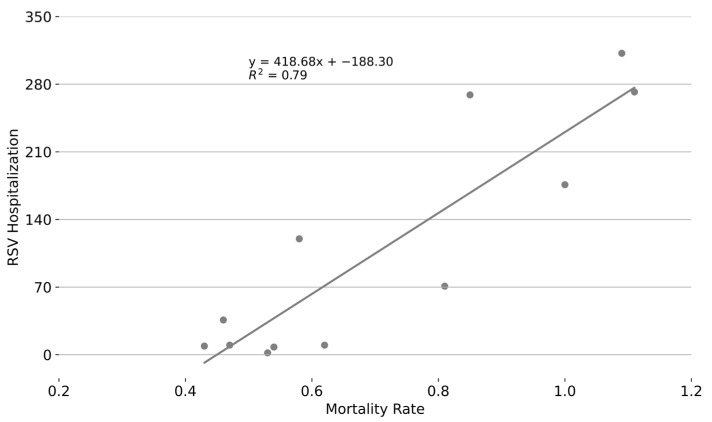
Correlation between monthly respiratory infant mortality rate in Mexico and total number of RSV-confirmed admissions of infants under one year of age in San Luis Potosí, Mexico.

To evaluate whether the seasonal distribution of respiratory infant mortality rates correlates with RSV activity, we analyzed the monthly number of RSV-confirmed ARI admissions of infants under one year of age based on epidemiological studies conducted between 2002 and 2015 in San Luis Potosí, Mexico. Figure 4 shows the monthly respiratory-associated infant mortality rates by month of occurrence alongside the number of RSV-confirmed hospital admissions for infants under one year of age in San Luis Potosí. The relationship between these variables was strong, with a coefficient of determination of *R*^2^ = 0.79, indicating that approximately 79% of the variance in respiratory infant mortality rates can be explained by RSV activity; see Figure 5.

**Figure 6:**
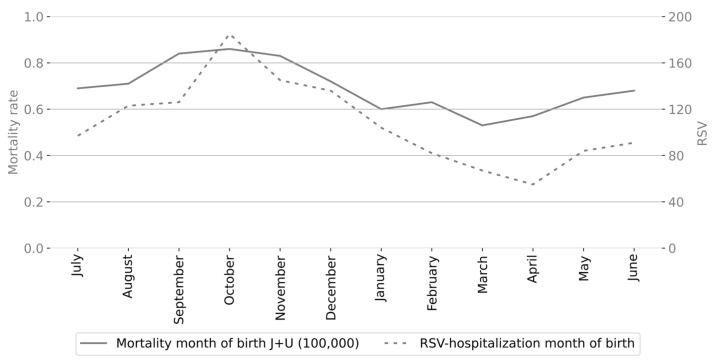
Monthly respiratory infant mortality rate in Mexico and total number of RSV-confirmed admissions of infants under one year of age in San Luis Potosí, Mexico based on the month of birth.

**Figure 7:**
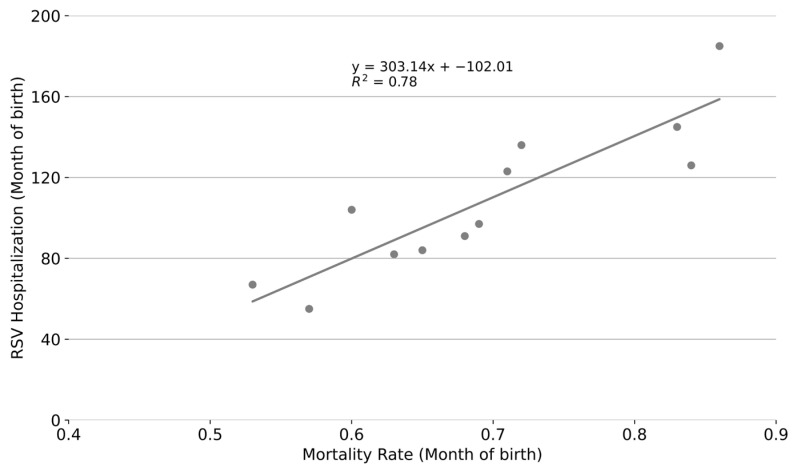
Correlation between monthly respiratory infant mortality rate in Mexico and total number of RSV-confirmed admissions of infants under one year of age in San Luis Potosí, Mexico based on the month of birth.

An analysis of respiratory infant mortality rates and RSV-related hospital admissions for infants under one year of age, based on their month of birth, revealed similar patterns. Infants born between September and December were hospitalized due to RSV more frequently than those born in other months, especially those born in March and April; see Figure 6. The relationship was quantitatively strong, with a coefficient of determination of *R*^2^ = 0.78, indicating that approximately 78% of the variance in respiratory infant mortality rates can be explained by RSV activity among infants under one year of age; see Figure 7.

### 3.3. Age of Death According to Month of Birth

**Figure 8:**
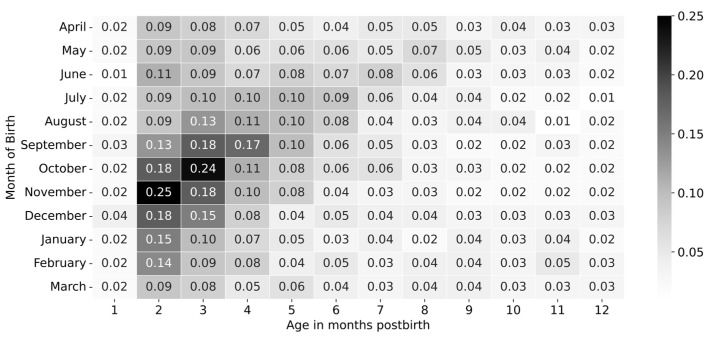
Mortality rate in each month of life for Mexican infants by month of birth. A comparison of mortality rates for infants born across the year is presented.

Utilizing mortality records with exact dates of birth and death (day, month, year), we analyzed the age distribution for respiratory deaths in our dataset. Overall, respiratory mortality rates were highest during the second month of life. However, differences emerged depending on the month of birth; see Figure 8. For infants born between November and June, the highest mortality rates occurred during the second month of life. In contrast, infants born between August and October experienced the highest mortality in the third month of life, while those born in July had the highest mortality during the fourth month. As infants grew older, mortality rates generally decreased, with the lowest rates occurring between the 10th and 12th months of life. However, infants born between January and May showed a slight increase in mortality towards the end of the first year of life. Overall, the highest mortality rates were observed during the second month of life for infants born in November (0.25 deaths per 1000 births) and during the third month for those born in October (0.24 deaths per 1000 births).

## 4. Discussion

Infant mortality varies between countries and within countries based on several demographic and healthcare characteristics [24,25]. In addition, seasonal variations in mortality have been recorded for a long time [26,27]. ARI-associated deaths contribute to seasonality in mortality, due to the epidemic behavior of major respiratory pathogens. Of note, while RSV is the main pathogen leading to severe ARI in infants, there are other important pathogens that contribute to the significant increase in ARI during the winter, such as influenza, SARS-CoV-2, and human metapneumovirus.

In this work, we carried out detailed analyses of respiratory infant mortality rates in Mexican children. Overall, respiratory infant mortality was highest for infants born in September, October, and November. In contrast, in the United States infants born between October and December were identified as those at highest risk of RSV-hospitalization during the first year of life [28], and those born between October and January had the highest RSV and bronchiolitis associated death rates [29]. In Croatia, infants born in November, December, and January were those at highest risk for ARI and RSV-associated hospitalizations [11]. While infants born in September, October, and November were those with higher respiratory rates in the present study, this varied in some regions, and mortality rates were also above the yearly average for infants born in August and December.

Detailed knowledge regarding the temporal behavior of infant mortality rates is useful in order to implement preventive strategies, such as immunization (active or passive) against RSV. In the international risk scoring tool, which stratifies late preterm infants at risk for severe RSV infection, birth between 3 months before and 2 months after the start of the virus season was identified as a factor associated with hospitalization [10]. In line with this, October has been identified as the month in which RSV circulation usually starts to increase in Mexico, and infants born between August and December, for whom respiratory mortality was above average, fall within this period.

Unfortunately, RSV detection is not carried out routinely in patients with respiratory tract infections, leading to a small number of RSV-specific diagnoses on hospital discharge records and in mortality registries in Mexico. Therefore, we used RSV-confirmed hospitalization cases from prospective epidemiological studies in San Luis Potosí to establish a correlation with monthly respiratory mortality. This was included to support the use of all respiratory deaths to assess RSV circulation (and help guide RSV preventive interventions). Few other studies lapsing more than one season have been carried out in Mexico and have also shown seasonal circulation of RSV [17,18]. While the RSV surveillance period used only partially overlapped with the mortality data in the study, surveillance data from RSV hospitalizations during the post-pandemic period suggests a stable seasonality of RSV infections.

In addition, RSV maternal immunization and long-acting monoclonal antibody administration programs rely on information of seasonal circulation of RSV for optimal application. For instance, in the United States, application of RSV vaccine in pregnant women is recommended starting in September (1–2 months before the anticipated start of RSV season), and continued through January (2–3 months before the anticipated end of the RSV season), while long-acting anti-RSV monoclonals are recommended for administration starting in October or November [30,31]. Given the wide geographical and climatological variations in Mexico, preventive programs may require regional adjustment to achieve higher effectiveness. Our results show that the seasonal increase in respiratory mortality rates starts between October and November in Mexico. However, seasonality tends to be less marked in some states. Of particular interest, the seasonality of respiratory mortality in Chiapas, Yucatán, and Morelos is not as notable as in most other states. Whether this reflects continuous circulation of viruses, such as RSV, should be determined, since this would have important implications on the seasonal administration of maternal immunization and long-acting monoclonal antibodies. In this context, local adjustments in the administration of anti-RSV monoclonal antibodies in some regions of Florida have been recommended due to the prolonged circulation of RSV which can even present year-round [32].

Several factors (such as maternal age, birth order, singleton vs. multiple pregnancies) have an impact on infant mortality [33]. We did not observe notable differences in maternal and paternal characteristics based on the month of birth, suggesting that viral epidemic circulation, meteorological conditions, or other factors that present with seasonal distribution were responsible for the observed differences in infant mortality between months.

Of interest, we observed different patterns for respiratory death age according to month of birth. While, in general, the highest mortality was recorded during the second month of life, mortality rates in infants born during the summer were highest in the third month of life. Li et al. have previously described differences in the age of highest risk for RSV hospitalization according to the month of birth [11]. In addition, our study provides information regarding regional differences within Mexico. These results suggest that taking into account the time and place of birth/residence could be valuable, together with individual information (such as underlying conditions, local environmental factors) for the institution of personalized and cost-effective preventive programs.

Limitations to our study include lack of information regarding the etiology of respiratory infections for most mortality records. Consequently, our analysis encompasses all respiratory-associated deaths rather than RSV-specific cases. However, the majority of respiratory mortality records are categorized under acute respiratory infection (ARI) codes, and RSV is widely recognized as the primary cause of severe respiratory infections in infants both globally and in Mexico. Given that ARI seasonal trends are largely driven by respiratory viruses—and that RSV has a higher case fatality rate than influenza and other respiratory viruses—our findings remain relevant for planning preventive programs targeting this virus [1,34,35].

Another limitation of the study is inclusion of the 2020–2021 season, with the addition of COVID-19-specific diagnostic codes which did not exist prior to 2020. Nevertheless, the number of COVID-19-associated deaths comprised slightly under 1% of all respiratory deaths. As such, the impact of inclusion of COVID-19 in the analysis is probably minimal. Of note, an important effect on the epidemiology and seasonality of respiratory viruses, including RSV, occurred during the COVID-19 pandemic, particularly between 2020 and 2022 [36]. It would be of interest to carry out follow-up studies once the pandemic has ended to assess the current impact of RSV on infant mortality, as well as the effect of future specific preventive strategies.

Additionally, despite using nationwide registries, we were unable to conduct analyses at the individual state level due to insufficient or missing monthly mortality data in some states. This limitation led us to aggregate states into broader regions. Although we based the regional classification on previously reported work, there might be differences within these regions that we may fail to capture due to aggregation of several states in each region. Nonetheless, the differences in peak and low mortality months across regions emphasize the value of conducting analyses at a sub-national level. Such regional distinctions suggest that immunization programs could benefit from tailoring strategies to enhance cost-effectiveness.

In conclusion, this study provides valuable insights into identifying high-risk infant groups for respiratory mortality in Mexico, based on both birth month and region of residence. This information can support more cost-effective implementation of preventive programs targeting specific respiratory viruses, particularly RSV. Although the results are specific to Mexico, the methodology used in this research can be applied in other countries.

## Figures and Tables

**Table 1:** Respiratory mortality diagnoses for Mexican infants born between April 2014 and March 2020 who died during the first year of life.

ICD-10 Codes	Diagnoses	Number of Deaths (*n* = 8805)	Percentage
J00X-J069	Upper respiratory tract infections	199	2.26
J09X-J118	Influenza	93	1.06
J120-J129	Viral pneumonia (including J12.1: RSV pneumonia)	54	0.61
J13X-J159	Bacterial pneumonia	282	3.2
J168-J189	Pneumonia by other agents and non-specified	4932	56.01
J200-J219	Bronchitis and bronchiolitis (including J21.0: acute bronchiolitis due to RSV)	545	6.19
J22X	Acute lower respiratory tract infection, non-specified	338	3.84
J304-J348	Rhinitis and other nasal disorders	8	0.09
J380-J399	Laryngeal, pharyngeal and other upper respiratory tract disorders	30	0.34
J42X-J449	Bronchitis, emphysema, and other chronic lung diseases	74	0.84
J450-J46X	Asthma	119	1.35
J677-J849	Pneumonitis associated with diverse conditions (including air humidifier, non-specified organic dusts, oils, among others) and other lung alveoli and interstitial disorders	624	7.09
J852-J948	Pulmonary abscess, pneumothorax and other pleural space disorders	95	1.08
J960-J969	Acute and chronic respiratory failure	171	1.94
J980-J989	Diverse tracheal, bronchial, and lung disorders	1153	13.09
U071-U072	COVID-19 ^1^	88	1

^1^ Coronavirus disease 2019.

**Table 2:** Respiratory infant mortality rates by birth month in Mexican children born between April 2014 and March 2020.

Month	East-Central	West-Central	South-Central	Northeast	Northwest	North	South	Yucatán Peninsula	National
July	0.69	0.51	0.85	0.52	0.67	0.64	0.95	0.78	0.69
August	0.74	0.59	0.73	0.36	0.75	0.77	0.97	0.70	0.71
September	0.80	0.73	1.02	0.52	0.91	0.86	1.02	1.03	0.84
October	0.96	0.71	0.86	0.52	0.72	0.79	1.05	1.22	0.86
November	0.84	0.71	0.92	0.63	0.79	0.93	0.93	0.88	0.83
December	0.72	0.62	0.86	0.58	0.78	0.60	0.98	0.60	0.72
January	0.49	0.52	0.83	0.51	0.52	0.59	0.95	0.73	0.60
February	0.52	0.45	0.75	0.39	0.69	0.64	1.11	0.84	0.63
March	0.43	0.42	0.45	0.36	0.32	0.62	1.08	0.62	0.53
April	0.51	0.50	0.65	0.45	0.55	0.41	0.96	0.45	0.57
May	0.57	0.51	0.77	0.49	0.44	0.50	1.21	0.77	0.65
June	0.69	0.47	0.70	0.58	0.60	0.58	1.03	1.03	0.68
Yearly Avg	0.67	0.57	0.79	0.50	0.66	0.67	1.02	0.82	0.70

Regional rates (deaths/1000 live births) represent the number of respiratory-related deaths per 1000 live births within each region and month.

## Data Availability

Only deidentified, publicly available registry data from INEGI were analyzed in this study, in full compliance with INEGI data use policies. Data supporting the results reported in this study can be found at https://github.com/AlessandroMilan/RespiratoryInfantMortality (accessed on 28 September 2025).

## Abbreviations

The following abbreviations are used in this manuscript:

RSV	Respiratory Syncytial Virus
INEGI	Instituto Nacional de Estadística y Geografía
ARI	Acute Respiratory Infections
ICD-10	International Classification of Diseases, Tenth Revision

## Appendix A

**Table A1:** Respiratory infant mortality rates by state in Mexican children born between April 2014 and March 2020.

State Code	State	Number of Births	Number of Deaths	Respiratory Mortality Rate *
1	Aguascalientes	154,704	80	0.52
2	Baja California	318,748	90	0.66
3	Baja California Sur	71,916	36	0.50
4	Campeche	94,852	58	0.61
5	Coahuila de Zaragoza	335,568	169	0.50
6	Colima	66,398	27	0.41
7	Chiapas	780,847	1093	1.40
8	Chihuahua	353,264	314	0.89
9	Ciudad de México	682,342	497	0.73
10	Durango	204,847	165	0.81
11	Guanajuato	669,729	429	0.64
12	Guerrero	425,281	242	0.57
13	Hidalgo	283,658	151	0.53
14	Jalisco	854,794	445	0.52
15	México	1,615,543	902	0.56
16	Michoacán de Ocampo	535,308	317	0.59
17	Morelos	177,192	57	0.32
18	Nayarit	117,962	66	0.56
19	Nuevo León	540,412	276	0.51
20	Oaxaca	447,348	349	0.78
21	Puebla	741,317	766	1.03
22	Querétaro	234,345	97	0.41
23	Quintana Roo	171,679	120	0.70
24	San Luis Potosí	287,471	161	0.56
25	Sinaloa	293,436	187	0.64
26	Sonora	265,796	202	0.76
27	Tabasco	265,659	228	0.86
28	Tamaulipas	331,702	157	0.47
29	Tlaxcala	143,860	119	0.83
30	Veracruz de Ignacio	739,486	570	0.77
31	Yucatán	209,705	211	1.00
32	Zacatecas	188,832	105	0.56
TOTAL	Mexico (Country)	12,604,902	8805	0.70

* Mortality rates represent the number of respiratory-related deaths per 1000 live births for each state over the period from 2014 to 2020.

**Table A2:** Classification of Mexican states into eight regions as used in the study.

Region *	States
North	Coahuila de Zaragoza, Chihuahua, Durango, San Luis Potosí, Zacatecas
Northwest	Baja California, Baja California Sur, Nayarit, Sinaloa, Sonora
Northeast	Nuevo Leon, Tamaulipas
West Central	Aguascalientes, Colima, Guanajuato, Jalisco, Michoacan de Ocampo
East Central	Ciudad de Mexico, Hidalgo, Mexico, Morelos, Puebla, Queretaro, Tlaxcala
South Central	Tabasco, Veracruz de Ignacio de la Llave
South	Chiapas, Guerrero, Oaxaca
Yucatán Peninsula	Campeche, Quintana Roo, Yucatan

* Each region groups states with geographical, climatological, and economical similarities to analyze regional differences in respiratory infant mortality rates.

**Table A3:** Monthly maternal and paternal characteristics of infants born during the study period.

Month	Total	Maternal Age (Median)	Paternal Age (Median)	Pregnancy Number (Median)	Singleton Pregnancy	Singleton Pregnancy (%)	Hospital Birth	Hospital Birth (%)	Hospital Birth (% from Specified) †	Single Mother	Single Mother (%)	Single Mother (% from Specified) ‡	Single, Divorced, Separated, Widow	Single, Divorced, Separated, Widow (%)	Single, Divorced, Separated, Widow (% from Specified) ‡
July	1,076,970	25	28	2	1,061,802	98.59	981,287	91.12	95.8	128,979	11.98	12.73	133,179	12.37	13.15
August	1,136,385	25	28	2	1,120,335	98.59	1,037,757	91.32	95.97	136,162	11.98	12.73	140,630	12.38	13.15
September	1,181,923	25	28	2	1,166,299	98.68	1,079,203	91.31	95.94	139,359	11.79	12.51	144,003	12.18	12.92
October	1,153,400	25	28	2	1,137,830	98.65	1,051,994	91.21	95.99	136,668	11.85	12.58	141,181	12.24	13
November	1,056,550	25	28	2	1,043,065	98.72	960,163	90.88	95.84	127,611	12.08	12.86	131,599	12.46	13.26
December	1,067,736	25	28	2	1,053,562	98.67	968,285	90.69	95.63	128,483	12.03	12.83	132,436	12.4	13.23
January	1,019,441	25	28	2	1,005,850	98.67	926,037	90.84	95.5	124,676	12.23	13.02	128,456	12.6	13.41
February	902,558	25	28	2	890,437	98.66	822,025	91.08	95.62	110,659	12.26	13.01	114,130	12.65	13.42
March	986,549	25	28	2	972,836	98.61	897,612	90.99	95.56	119,649	12.13	12.88	123,348	12.5	13.28
April	994,946	25	28	2	981,828	98.68	904,015	90.86	95.42	117,510	11.81	12.51	121,299	12.19	12.92
May	1,024,246	25	28	2	1,010,753	98.68	932,777	91.07	95.56	121,184	11.83	12.53	125,060	12.21	12.93
June	1,004,198	25	28	2	990,491	98.64	915,144	91.13	95.71	119,410	11.89	12.61	123,340	12.28	13.03

† Birth place not specified: 615,775 (4.89%). ‡ Marital status not specified: 737,473 (5.85%).

**Table A4:** Monthly maternal and paternal educational level characteristics of infants born during the study period.

Month	Total	Mother Without Any Education Level	Mother Without Any Education Level (%)	Mother Without Any Education Level (% from Specified) †	Mother with Elementary Education	Mother with Elementary Education (%)	Mother with Elementary Education (% from Specified) †	Mother with Elementary Education or No Educational Level	Mother with Elementary Education or No Educational Level (%)	Mother with Elementary Education or No Educational Level (% from Specified) †	Father Without Any Education Level	Father Without Any Education Level (%)	Father Without Any Education Level (% from Specified) ‡	Father with Elementary Education	Father with Elementary Education (%)	Father with Elementary Education (% from Specified) ‡	Father with Elementary Education or No Educational Level	Father with Elementary Education or No Educational Level (%)	Father with Elementary Education or No Educational Level (% from Specified) ‡
July	1,076,970	20,401	1.89	2.03	183,036	17.0	18.21	203,437	18.89	20.24	19,625	1.82	2.14	187,230	17.38	20.4	206,855	19.21	22.54
August	1,136,385	21,375	1.88	2.01	190,590	16.77	17.96	211,965	18.65	19.97	20,751	1.83	2.14	195,167	17.17	20.14	215,918	19.0	22.28
September	1,181,923	22,567	1.91	2.04	197,797	16.74	17.9	220,364	18.64	19.94	21,984	1.86	2.17	203,421	17.21	20.1	225,405	19.07	22.28
October	1,153,400	22,212	1.93	2.06	191,135	16.57	17.75	213,347	18.5	19.82	21,631	1.88	2.19	196,657	17.05	19.95	218,288	18.93	22.15
November	1,056,550	20,782	1.97	2.11	176,192	16.68	17.89	196,974	18.64	20.0	20,197	1.91	2.24	181,877	17.21	20.22	202,074	19.13	22.46
December	1,067,736	21,404	2.0	2.15	176,522	16.53	17.77	197,926	18.54	19.92	20,685	1.94	2.28	182,922	17.13	20.15	203,607	19.07	22.42
January	1,019,441	20,114	1.97	2.12	170,321	16.71	17.92	190,435	18.68	20.03	19,445	1.91	2.24	174,394	17.11	20.1	193,839	19.01	22.34
February	902,558	17,534	1.94	2.08	150,097	16.63	17.81	167,631	18.57	19.89	17,072	1.89	2.22	154,890	17.16	20.12	171,962	19.05	22.34
March	986,549	18,890	1.91	2.05	165,146	16.74	17.93	184,036	18.65	19.98	18,172	1.84	2.16	169,659	17.2	20.19	187,831	19.04	22.35
April	994,946	19,461	1.96	2.09	175,816	17.67	18.91	195,277	19.63	21.0	18,373	1.85	2.17	179,252	18.02	21.13	197,625	19.86	23.3
May	1,024,246	19,804	1.93	2.07	179,654	17.54	18.75	199,458	19.47	20.82	18,735	1.83	2.14	182,518	17.82	20.88	201,253	19.65	23.02
June	1,004,198	19,428	1.93	2.07	173,344	17.26	18.47	192,772	19.2	20.54	18,270	1.82	2.13	175,962	17.52	20.54	194,232	19.34	22.67

† Unknown maternal educational level: 838,813 (6.65%). ‡ Unknown paternal educational level: 1,855,792 (14.72%).
